# Supplementary material for: Visual Perceptual Learning of Form–Motion Integration: Exploring the Involved Mechanisms with Transfer Effects and the Equivalent Noise Approach
Source: Brain Sci. 2024 Sep 30;14(10):997. doi: 10.3390/brainsci14100997 (PMC11506814; doi:10.3390/brainsci14100997)
Supplement: Supplementary file 1 [file brainsci-14-00997-s001.zip › brainsci-3222695-supplementary.pdf]

Supplementary material

Video S1. (**a**) Illustrates the dynamic GPs, where dipoles appear to shift along the vertical axis, creating an illusion of directional motion despite the absence of coherent motion (i.e., no dipole-to-dipole correspondence between successive patterns) (see https://osf.io/p68tf). (**b**) Shows the mRDKs, depicting randomly distributed dots drifting along the vertical axis. In both videos, two temporal intervals are presented: the first interval always contains the coherent pattern (100% coherence), while the second interval always contains a random/noise pattern (GPs and mRDKs) (see https://osf.io/m2f36). However, in the actual experiment, the temporal intervals with the coherent non-directional motion and the noise pattern were presented in random order.
